# A Case of Omeprazole-Associated Acute Interstitial Nephritis

**DOI:** 10.7759/cureus.55035

**Published:** 2024-02-27

**Authors:** Jakob Nypaver, Devi Nair, Sujay Deshpande, Shefali Amin, Jenna Wynn, Manish Shrestha, William Pompella

**Affiliations:** 1 Medicine, Drexel University College of Medicine, Philadelphia, USA; 2 Internal Medicine, Tower Health Medical Group, Reading, USA

**Keywords:** internal medicine, clinical nephrology, medication side effects, renal pathology, proton pump inhibitor, acute interstitial nephritis

## Abstract

Acute interstitial nephritis (AIN) is characterized by an inflammatory infiltrate of the interstitium of the kidney, typically causing a decline in kidney function. Drug-induced AIN (also called allergic AIN) is a type of AIN. Common drugs associated with AIN are antibiotics, non-steroidal anti-inflammatory drugs (NSAIDs), and proton pump inhibitors (PPIs). A 59-year-old male with a history of recent laparoscopic robotic sleeve gastrectomy presented to the emergency department with five weeks of progressively worsening fatigue, nausea, and lightheadedness. Postoperatively, he was prescribed omeprazole 20 mg daily for gastric ulcer prophylaxis. His other home medications were amlodipine, atorvastatin, ursodiol, and budesonide-formoterol fumarate nebulizer. His physical examination was normal. Laboratory studies revealed elevated creatinine of 4.19 mg/dL from a baseline of 0.9 mg/dL two months ago and the presence of urine eosinophils. The etiology of this elevated creatinine was unclear, prompting CT-guided left renal biopsy. The biopsy showed diffuse interstitial inflammatory infiltration with numerous lymphocytes, a large number of neutrophils, and scattered eosinophils, consistent with the allergic type of AIN. Omeprazole was discontinued and the patient received a seven-day course of prednisone. Despite treatment, permanent renal damage occurred, and the patient’s new baseline creatinine was 2.3 mg/dL. AIN caused by PPIs should be considered in the differential diagnosis of acute kidney injury (AKI). AIN can be difficult to diagnose, presenting with nonspecific symptoms, such as oliguria, malaise, nausea, and vomiting. An accurate and timely diagnosis can help prevent and treat worsening renal failure.

## Introduction

Acute interstitial nephritis (AIN) is a condition that can cause acute kidney injury (AKI). AIN is characterized by an inflammatory infiltrate of the kidney interstitium, typically leading to a decline in kidney function [1]. Drug-induced AIN (DI-AIN), also called allergic AIN, is the most common cause of AIN in developed countries [2]. Common drugs associated with AIN include non-steroidal anti-inflammatory drugs (NSAIDs), proton pump inhibitors (PPIs), antibiotics (including penicillins, cephalosporins, rifampin, sulfonamides, and ciprofloxacin), and more [2]. NSAIDs and antibiotics account for 44% and 33% of DI-AIN, respectively [2].

A classic clinical presentation of drug-induced AIN involves the presence of a fever, rash, and eosinophilia occurring within days to weeks after initiating the offending drug [3]. PPIs are not the most common cause of DI-AIN, but AIN is a possible side effect. The prevalence of AIN in omeprazole was six per 10,000 cases [4]. In renal biopsy performed for an unexplained AKI, DI-AIN was present in 20% of biopsies [2].

PPIs are considered first-line therapies for most pathologies associated with elevated acid secretion in the stomach lining including, but not limited to, peptic ulcers, *Helicobacter pylori*-related pathology, and gastroesophageal reflux disease [5]. In addition, PPIs have off-label uses, mainly related to gastritis prophylaxis. Because of this, PPIs are some of the most widely used acid suppression medications worldwide [5]. Indications for long-term PPI use include Zollinger-Ellison syndrome and Barrett’s esophagus; however, for most other conditions, PPI use is recommended to be limited to four to eight weeks [5]. Despite recommendations, the long-term use of PPIs is becoming more prevalent [5]. A case-controlled study in New Zealand found that patients diagnosed with AIN secondary to PPI use are at a significantly increased risk for hospitalization and death [6]. We report a case of AIN resulting from PPI use following a recent surgical procedure.

This case report was previously presented at the American College of Physicians (ACP) Pennsylvania Southeastern Region as a poster presentation on October 7, 2023.

## Case presentation

A 59-year-old male with a past medical history of hypertension, dyslipidemia, chronic obstructive pulmonary disease, obstructive sleep apnea on continuous positive airway pressure, and recent laparoscopic robotic sleeve gastrectomy and hiatal hernia repair for weight loss presented to the emergency department with five weeks of progressively worsening fatigue, nausea, and lightheadedness. He underwent sleeve gastrectomy about eight weeks prior to admission, where he received 900 mg of clindamycin for his preoperative antibiotics. Postoperatively, he was prescribed omeprazole 20 mg oral daily for gastric ulcer prophylaxis, in addition to his home medications that included amlodipine, atorvastatin, ursodiol, and budesonide-formoterol fumarate nebulizer. A day prior to admission, the patient had blood work performed as a part of his outpatient follow-up. The blood work revealed abnormalities, including a creatinine of 3.9 mg/dL (from the patient’s previous baseline of 0.9 mg/dL prior to surgery) and hemoglobin of 9.4 g/dL (from the patient’s previous baseline of 13-14 g/dL prior to surgery).

The patient was hemodynamically stable and afebrile. His physical examination, including orthostatic vital signs, was unremarkable. Skin examination showed no rash. Laboratory values from day one of his admission are listed in Table 1. Notably, there was an elevated creatinine of 4.19 mg/dL. Urine analysis with microscopic was notable for 150 mg/dL glucose, 10 mg/dL ketones, 100 mg/dL protein, and 3+ hyaline casts. A urine eosinophil was ordered, which showed a few eosinophils. Renal ultrasound and duplex were unremarkable, demonstrating non-atrophic kidneys bilaterally. Computed tomography (CT) without contrast of the abdomen and pelvis revealed no renal calculi, hydronephrosis, or ureteral calculus. Initially, a pre-renal AKI was suspected due to extreme dietary restriction and decreased oral intake. The urine sodium level was 77 mmol/L; however, due to extreme dietary restriction and lightheadedness, fluid resuscitation was the initial treatment. The following day the creatinine mildly improved to 4.05 mg/dL, and then progressively declined.

**Table 1:** Admission laboratory values BUN: blood urea nitrogen, WBC: white blood cell, RBC: red blood cell, ANA: antinuclear antibody, ANCA: antineutrophil cytoplasmic antibody, IgM: immunoglobulin M

Parameters	Patient values	Reference range
Sodium	138 mmol/L	136-145 mmol/L
Potassium	3.6 mmol/L	3.5-5.1 mmol/L
Chloride	107 mmol/L	98-107 mmol/L
CO_2_	21.1 mmol/L	21.0-31.0 mmol/L
Glucose	115 mg/dL	70-99 mg/dL
Creatinine	4.19 mg/dL	0.60-1.30 mg/dL
BUN	41 mg/dL	7-25 mg/dL
Calcium	9.2 mg/dL	8.6-10.3 mg/dL
Anion gap	10 mmol/L	5-12 mmol/L
WBC	7.1 10E3/uL	4.8-10.8 10E3/uL
RBC	3.05 10E6/uL	4.50-6.10 10E6/uL
Hemoglobin	9.0 g/dL	14.0-17.5 g/dL
Hematocrit	28.5%	39.0-53.0%
Platelets	288 10E3/uL	130-400 10E3/uL
Eosinophil count	0.39 10E3/uL	0.04-0.54 10E3/uL
C3 level	113 mg/dL	82-185 mg/dL
C4 level	38.4 mg/dL	15.0-53.9 mg/dL
ANA screen	Negative	
ANCA screen	Negative	
IgM	381 mg/dL	50-300 mg/dL
Kappa-free light chain	89.9 mg/dL	3.3-19.4 mg/L
Kappa/Lamba-free light chain ratio	3.76	0.26-1.65

**Figure 1:**
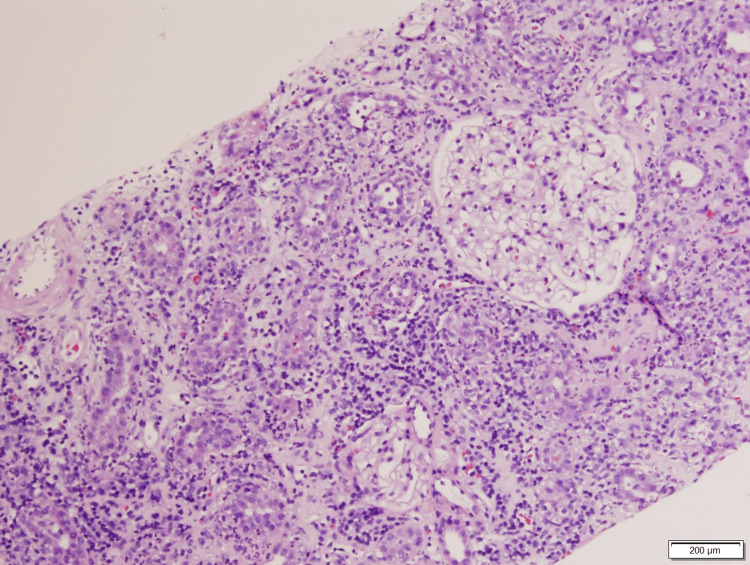
Hematoxylin and eosin stain. Glomeruli are normal with open loops and smooth capillaries. The background shows diffuse interstitial edema with inflammatory infiltration.

**Figure 2:**
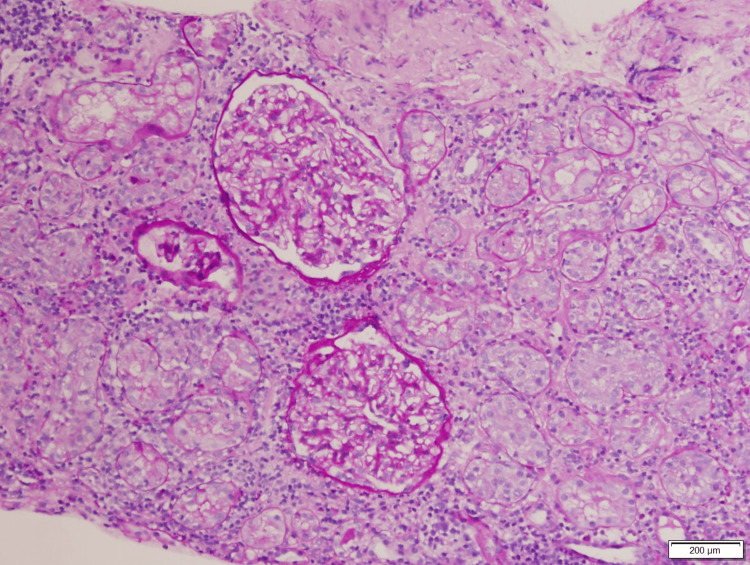
Periodic acid-Schiff stain. Intact basement membrane without pathology. As in Figure 1, glomeruli are normal with open loops and smooth capillaries.

**Figure 3:**
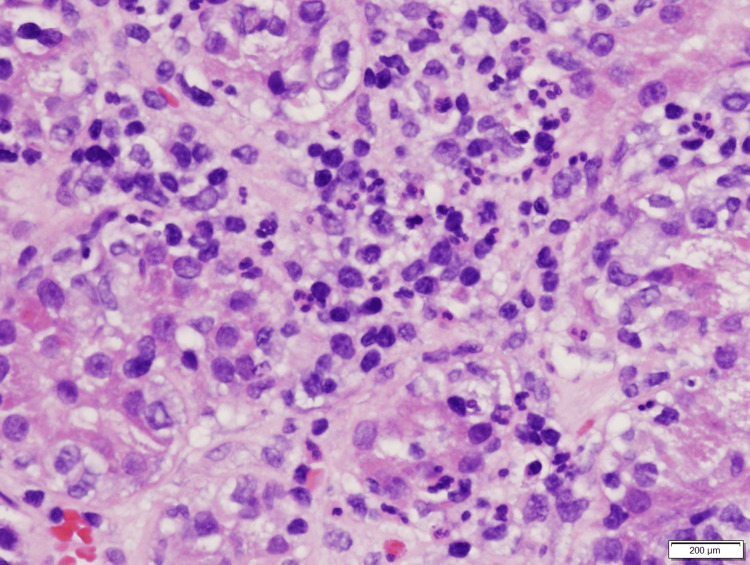
Inflammatory reaction with lymphocytes, neutrophils, and rare eosinophils.

**Figure 4:**
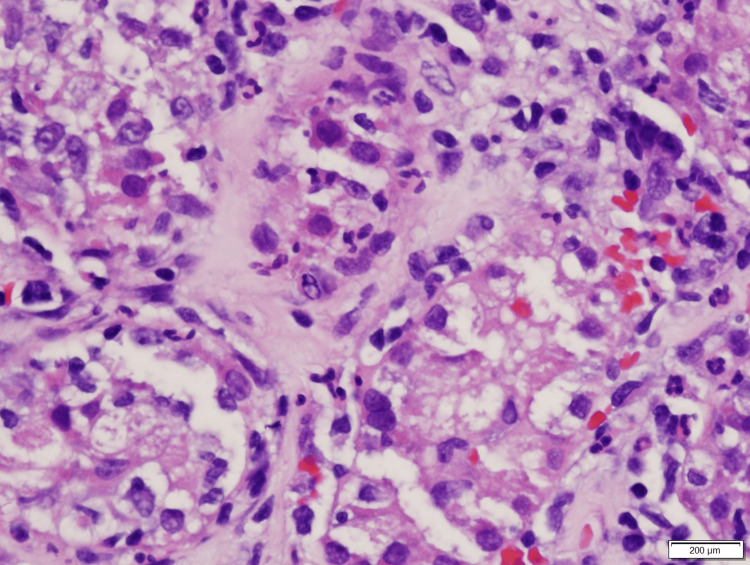
Focal tubulitis and neutrophils seen in the tubules with epithelial cell injury.

At this point, an intrarenal AKI was suspected, but the etiology was unclear. A CT-guided left renal biopsy was performed (Figures 1-4), which revealed diffuse interstitial inflammatory infiltration with numerous lymphocytes, a large number of neutrophils, and scattered eosinophils, consistent with an allergic type of AIN. No glomerular injury was seen, but tubular injury and focal tubulitis were also noted. Omeprazole was discontinued and added as an allergy in his medical record. The patient received an initial IV dose of methylprednisolone 40 mg and transitioned to oral prednisone 40 mg daily with improvement in his symptoms. He was discharged home to complete a seven-day course of steroids and plan for outpatient follow-up with his primary care provider. Despite appropriate treatment with steroids, the patient’s kidney function did not return to his pre-operative baseline. One month after treatment, his new baseline creatinine was 2.3 mg/dL.

## Discussion

This case depicts a well-known idiosyncratic adverse effect of PPIs. The first case of omeprazole-associated acute interstitial nephritis was reported by Ruffenanch et al. in 1992 [7]. AIN can be challenging to diagnose, and many cases are asymptomatic. The cause of this patient’s AKI was not clear until the biopsy results. There are a few points to address in the diagnosis of this case.

The differential diagnosis of AKI includes pre-renal, intrarenal, and post-renal causes of injury. All three causes will take serum creatinine and blood-urea-nitrogen levels into account. The physical exam and history of pre-renal AKI are important, with attention to findings of volume depletion [1]. A pre-renal AKI will respond to volume repletion. In this case, the patient was on dietary restrictions following gastric surgery, and the complaint of lightheadedness prompted the initial thought of a pre-renal AKI. The patient’s creatinine showed minimal improvement after receiving IV fluids but proceeded to increase the following day. Furthermore, a post-renal AKI is usually caused by an obstruction that can be identified by imaging [3]. After ruling out pre- and post-renal causes, an intrinsic cause of AKI was suspected. However, the underlying cause of this patient’s intrinsic injury was not clear.

Possible causes of intrinsic AKIs include AIN, acute tubular necrosis (ATN), and glomerular disease. In order to determine the etiology, laboratory testing and, in some cases, biopsy are performed. A biopsy is not always necessary [1], as stopping the offending agent and monitoring response to treatment can be adequate. A biopsy establishes a definitive diagnosis, but it often does not change the treatment plan since clinical suspicion often guides therapy [2]. However, if the cause of declining renal function is unclear, as in this case, a biopsy can be performed to aid in the diagnosis.

Urinalysis, urinary protein excretion, basic metabolic panel, and complete blood count are some initial tests ordered. Special attention should be paid to urine and serum eosinophil levels. Eosinophiluria was at one time considered pathognomonic for AIN. While still a good diagnostic clue, recent data suggest that it lacks the sensitivity and specificity needed for diagnosis [8].

This patient’s etiology of AIN was likely a result of the recently prescribed PPI. The World Health Organization Uppsala Monitoring Centre (WHO-UMC) causality category term for this case is "probable/likely." The rationale for the causality term is the abnormal laboratory test occurred within a reasonable time relationship to drug intake; this adverse event was unlikely to be attributed to disease or other drugs, and the response to withdrawal was clinically reasonable [9]. Today, PPI use is widespread and can be obtained with a prescription or over the counter [5]. Moreover, consideration should be taken by physicians to avoid a reflexive pattern of prescribing PPIs and review PPI use with the aim of deprescribing when clinical indication has dissipated [5].

## Conclusions

AIN can be difficult to diagnose, presenting with nonspecific symptoms, such as skin rash, oliguria, malaise, nausea, and vomiting. An accurate and timely diagnosis can help prevent permanent renal damage. AIN is a known adverse effect of PPIs and should be considered in the differential diagnosis of an AKI patient on medications known to cause drug-induced AIN. Medication reconciliation could have helped identify the recent addition of omeprazole to this patient’s medications. Careful consideration should be taken when prescribing PPIs, and providers should be careful not to reflexively prescribe PPIs as prophylaxis.
